# Piperazine and Morpholine Protoporphyrin IX Derivatives Intensify Biomolecules Oxidation and Phototoxicity in Triple‐Negative Breast Cancer

**DOI:** 10.1002/cmdc.70471

**Published:** 2026-08-28

**Authors:** Maynne Duarte Suriani Franco, Thibault Joseph William Jacques Dit Lapierre, Tarcisio Paiva Mendonça, Luiz Felipe Fernandes Peixoto, Renata Roland Teixeira, Joyce Ferreira Da Costa Guerra, Kelly Aparecida Geraldo Yoneyama Tudini, Daniele Lisboa Ribeiro, Foued Salmen Espindola, Allisson Benetti Justino, Celso de Oliveira Rezende Júnior, Tayana Mazin Tsubone

**Affiliations:** ^1^ Institute of Chemistry Federal University of Uberlandia Uberlandia Minas Gerais Brazil; ^2^ Institute of Biotechnology Federal University of Uberlandia Uberlandia Minas Gerais Brazil; ^3^ Institute of Biomedical Sciences Federal University of Uberlandia Uberlandia Minas Gerais Brazil; ^4^ Department of Chemistry Federal University of Juiz de Fora Juiz de Fora Minas Gerais Brazil

**Keywords:** breast cancer, morpholine, photodynamic activity, piperazine, protoporphyrin IX

## Abstract

Photodynamic therapy (PDT) is a clinically established approach that relies on the localized generation of reactive oxygen species (ROS) mediated by a photosensitizer (PS), light, and molecular oxygen. Protoporphyrin IX (PpIX) is an approved PS; however, its intracellular retention is limited, factor that can alter its overall photodynamic efficiency. In this study, we report how the design of PpIX derivatives bearing piperazine (PpIX‐Pip) or morpholine (PpIX‐Morp) substituents may modulate lipophilicity, cellular uptake, oxidative stress, and phototoxicity. The photodynamic activity of these derivatives was evaluated in the aggressive triple‐negative human breast cancer cell line MDA‐MB‐231. Both derivatives showed greater time‐dependent cellular retention and enhanced photodynamic activity compared to PpIX, promoting oxidative stress, and triggering loss of mitochondrial membrane potential and caspase‐3 activation. These results highlight the impact of rational structural modification of PpIX on photodynamic efficiency and provide valuable structure–activity relationship insights for the development of improved porphyrin‐based PS for PDT.

## Graphical abstract

Protoporphyrin IX derivatives bearing piperazine or morpholine substituents exhibit enhanced cellular retention and photodynamic activity in triple‐negative breast cancer cells. Structural modification promotes oxidative stress, alters mitochondrial membrane potential, and increases caspase‐3 activity, providing new structure–activity relationship insights for the rational design of improved porphyrin‐based photosensitizers.

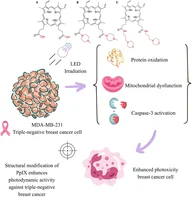

## 1 Introduction

Photodynamic therapy (PDT) is a therapeutic modality that combines a photosensitizer (PS), light of an appropriate wavelength, and molecular oxygen to generate cytotoxic reactive oxygen species (ROS) for the treatment of oncological diseases [1, 2, 3, 4, 5]. Compared to conventional cancer therapies, PDT offers several advantages, including reduced invasiveness, high spatial selectivity, minimal systemic toxicity, and the possibility of repeated treatments at the same site [1, 6]. These features have driven sustained efforts toward the development of optimized photosensitizers with improved pharmacological and photophysical profiles.

The efficacy of PDT critically depends on the physicochemical and biological properties of the PS, including its absorption characteristics, singlet oxygen quantum yield, cellular uptake, subcellular localization, and phototoxicity [7, 8]. Upon light absorption, PS molecules are promoted to excited singlet states and may undergo intersystem crossing to long‐lived triplet states, which interact with molecular oxygen to generate ROS [9]. Owing to the short lifetime and limited diffusion distance of these species, effective PDT requires localization of the PS in close proximity to key cellular targets, such as membranes and mitochondria, where oxidative damage can efficiently trigger programmed cell death pathways, particularly apoptosis [5, 10].

Porphyrins and their derivatives are among the most extensively investigated classes of photosensitizers, owing to their favorable photophysical properties, structural versatility, and intrinsic affinity for tumor tissues [11, 12, 13]. Among them, protoporphyrin IX (PpIX) is clinically approved and widely used; however, its therapeutic performance is limited by its poor solubility in physiological environments and a pronounced tendency to aggregate, which negatively impacts singlet oxygen generation and overall photodynamic efficiency [14]. Consequently, structural modification of PpIX has emerged as an effective strategy to overcome these limitations and enhance PDT outcomes [15, 16].

Chemical derivatization of PpIX enables fine‐tuning of key molecular parameters such as lipophilicity, amphiphilicity, membrane permeability, and biological selectivity [17, 18]. Lipophilicity is a central concept in medicinal chemistry and plays a decisive role in absorption, distribution, metabolism, excretion, and toxicity (ADMET) profiles [19]. Achieving a balanced amphiphilic character is therefore essential for the rational design of efficient PDT agents. In this context, the incorporation of positively charged substituents can enhance electrostatic interactions with negatively charged cellular membranes, promoting intracellular retention and localized ROS generation [20, 21].

Piperazine and morpholine are privileged scaffolds in medicinal chemistry and are widely employed to improve pharmacokinetic properties, membrane interactions, and anticancer activity [22, 23, 24, 25, 26, 27, 28]. Porphyrin–piperazine conjugates have demonstrated enhanced antiproliferative activity against several tumor cell lines, including hepatoma, nasal carcinoma, and gastric cancer models, potentially due to strengthened interactions with biomolecules such as DNA [14, 15, 16, 17, 18]. Similarly, morpholine‐containing photosensitizers have shown improved photophysical properties and PDT efficacy, which have been attributed to enhanced interactions with proteins and nucleic acids, resulting in efficient photo‐induced oxidative damage and cell death [29, 30, 31]. Despite these promising findings, systematic investigations of the photodynamic activity of PpIX derivatives bearing piperazine or morpholine substituents remain limited [9, 19, 20, 21, 32].

**FIGURE 1:**
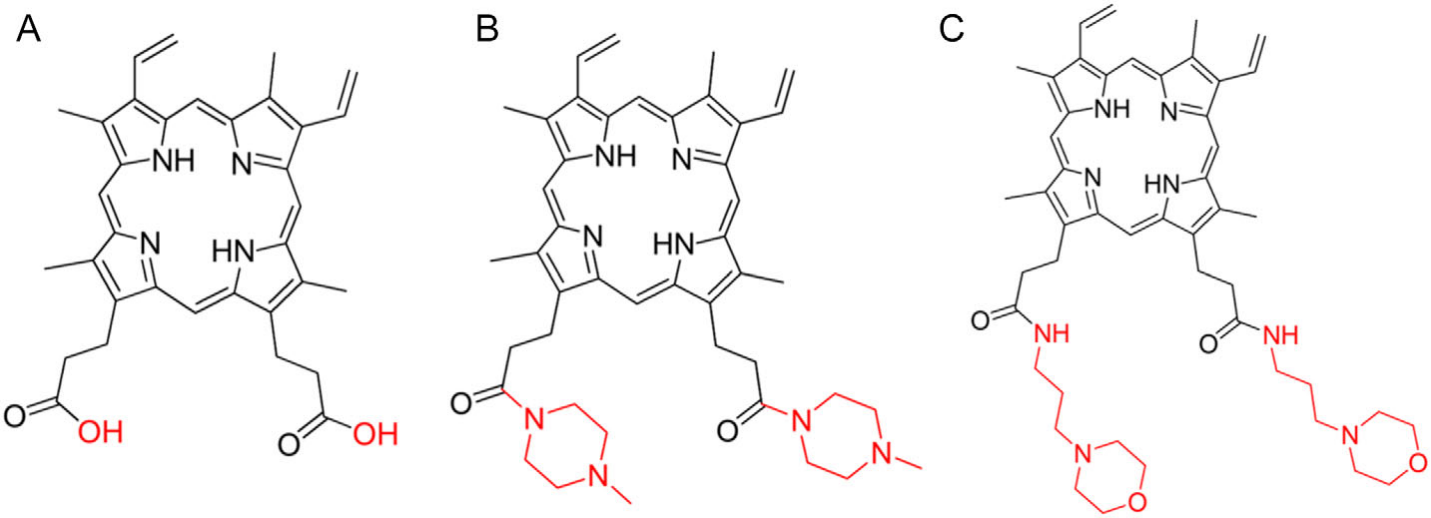
Chemical structures of (A) protoporphyrin IX (PpIX) and its derivatives: (B) PpIX–methyl‐piperazine (PpIX‐Pip) and (C) PpIX–morpholine (PpIX‐Morp).

Previously, we reported the synthesis and physicochemical characterization of protoporphyrin IX (PpIX—Figure 1A) diamide derivatives functionalized with piperazine (PpIX‐Pip—Figure 1B) and morpholine (PpIX‐Morp—Figure 1C), including an evaluation of their structural and photophysical properties, as well as photodynamic activity against HeLa cells [33]. Building on these findings, the present study aims to further investigate how these structural modifications (Figure 1) influence key biological responses relevant to PDT. Specifically, we assess the impact of these derivatives on lipophilicity, cellular interaction, photodynamic efficiency, reactive oxygen species (ROS) generation, oxidative stress, mitochondrial dysfunction, and apoptotic signaling—particularly caspase‐3 activation—in the aggressive triple‐negative human breast cancer cell line MDA‐MB‐231 [5, 10]. By extending our previous work, these results contribute to a deeper understanding of structure–activity relationships, supporting the rational development of improved porphyrin‐based photosensitizers for PDT.

## 2 Experimental Section

### 2.1 Chemicals and Reagents

All chemicals and reagents were of analytical or HPLC grade and used as received without further purification. Dimethyl sulfoxide (DMSO, CAS 67‐68‐5, Synth), n‐octanol (CAS 111‐87‐5, Vetec), and absolute ethanol (CAS 64‐17‐5, *Dinamica*) were used as solvents. Hydrogen peroxide (H_2_O_2_), Dulbecco's Modified Eagle Medium (DMEM), 2′,7′‐dichlorodihydrofluorescein diacetate (DCFH‐DA), rhodamine 123 (CAS 62 669‐70‐9), 5,5‐dithiobis(2‐nitrobenzoic acid) (DTNB), and pyrogallol were purchased from *Sigma–Aldrich*. Fetal bovine serum (FBS), penicillin–streptomycin, and trypsin–EDTA were obtained from *Vitrocell Embriolife*. Thiazolyl blue tetrazolium bromide (MTT, CAS 298‐93‐1, Ambeed) and neutral red (NR) were used as cell viability indicators.

Phosphate‐buffered saline (PBS) and PBS–KCl buffer (20 mM phosphate, 140 mM KCl, pH 7.4) were prepared using analytical‐grade reagents and stored at 4 °C prior to use.

### 2.2 Synthesis, Purification, and Characterization of PpIX Derivatives

PpIX‐Pip (Figure 1B) and PpIX‐Morp (Figure 1C) were synthesized from PpIX through EDC and HOBt mediated amidation reactions, as previously reported [33]. PpIX‐Pip was obtained in 93% yield and PpIX‐Morp in 39% yield. Their identities were confirmed by ^1^H NMR, ^13^C NMR and HRMS [33].

### 2.3 Determination of the Partition Coefficient (Log P)

The partition coefficient (Log P) was determined using the shake‐flask method recommended by the Organization for Economic Co‐operation and Development (OECD) [19]. Owing to the limited solubility of porphyrins in the *n*‐octanol/water system, 1 mM stock solutions were prepared in DMSO and vortexed until complete dissolution.

Subsequently, 2 mL of water and 2 mL of *n*‐octanol were added to a glass flask, followed by the addition of 40 µL of the stock solution to form the biphasic system. The mixture was vortexed for 10 min and then allowed to stand for 24 h to ensure complete phase separation. Aliquots (200 µL) from each phase were collected and diluted in 2 mL of DMSO prior to absorbance measurements using a UV–Vis spectrophotometer (Shimadzu, UV‐2501‐PC) with a 1.0 cm path‐length cuvette. Log *p* values were calculated according to Equation ((1)) [19, 34].

(1) $$\text{LogP}=\log\left(\frac{A_{n-\text{octanol}}}{A_{\text{water}}}\right)$$

where LogP is partition coefficient, (*A*_*n*−octanol_) is absorbance value at *λ*_max_ (401 nm) of the compound in n‐octanol, and (*A*_water_) is absorbance value at *λ*_max_ (401 nm) of the compound in water.

### 2.4 Cell Culture

Human triple‐negative breast adenocarcinoma MDA‐MB‐231 cells (ATCC HTB‐26, RRID:CVCL_0062) and nontumorigenic human mammary epithelial MCF‐10A cells (ATCC CRL‐10 317, RRID:CVCL_0598), obtained from the American Type Culture Collection (ATCC, Manassas, VA, USA), were maintained under standard cell culture conditions. MDA‐MB‐231 cells were cultured in Dulbecco's Modified Eagle Medium (DMEM) containing phenol red and supplemented with 10% (v/v) fetal bovine serum (FBS) and 1% (v/v) penicillin–streptomycin (100 U mL^−1^ penicillin and 100 μg mL^−1^ streptomycin). MCF‐10A cells were cultured in DMEM/F12 supplemented with 5% (v/v) FBS, 20 ng mL^−1^ epidermal growth factor (EGF), 10 μg mL^−1^ insulin, 0.5 μg mL^−1^ hydrocortisone, and 1% (v/v) penicillin–streptomycin. Both cell lines were maintained at 37 °C in a humidified atmosphere containing 5% CO_2_. Cells in the exponential growth phase were routinely subcultured by removing the culture medium, washing with phosphate‐buffered saline (PBS), and incubating with 0.25% trypsin–EDTA for 2 min. Trypsinization was terminated by the addition of fresh complete culture medium, and cells were passaged twice weekly.

### 2.5 Cellular Uptake of PpIX Derivatives in Breast Cancer Cells

MDA‐MB‐231 cells were seeded in 12‐well plates at a density of 1.5 × 10^5^ cells per well in 1 mL of culture medium and incubated for 24 h to allow cell adhesion. Photosensitizer solutions were prepared by diluting the DMSO stock solutions in supplemented DMEM to a final concentration of 5 µM, ensuring that the final DMSO amount did not exceed 1% (v/v). After cell attachment, cells were incubated with each porphyrin (1 mL per well) for 1, 3, or 24 h in the dark at 37 °C under a 5% CO_2_ atmosphere. After incubation time, 1 mL of the supernatant (containing noninternalized PS) was collected and diluted with 2 mL of DMSO. Adherent cells were washed with PBS and lysed with 2 mL of DMSO to extract the internalized PS, followed by dilution with 1 mL of supplemented DMEM.

Absorption spectra in the band area 401 nm of both supernatant and cell lysate samples were recorded using a UV–Vis spectrophotometer (PerkinElmer, EnSpire). The percentage of cellular uptake was calculated using Equation ((2)) [35].

(2) $$\text{\% Uptake}=\frac{{\mathrm{Abs}}_{\text{cell lysate}}}{{\mathrm{Abs}}_{\text{cell lysate}}\;+\;{\mathrm{Abs}}_{\text{supernatant}}}$$

where Abs_cell_ _lysate_ is the absorbance band area of the porphyrin extracted from the cell (incorporated PS), and Abs_supernatant_ is the absorbance band area of the porphyrin in supernatant solution (nonincorporated PS).

### 2.6 Cell Viability via MTT and Neutral Red Method

MDA‐MB‐231 and MCF‐10A cells were seeded in 96‐well plates at a density of 2.5 × 10^4^ cells per well and incubated for 24 h at 37 °C in a 5% CO_2_ atmosphere to allow complete adhesion. The culture medium was then replaced with fresh medium containing the PSs at concentrations ranging from 45 to 300 nM.

After 24 h of incubation with the PSs, cells were washed with PBS (pH 7.4) to remove noninternalized compounds. One plate was maintained in the dark as a control, while the remaining plates were irradiated using white LEDs positioned 7 cm above the plate, delivering a light dose of 3.0 J cm^−2^ (i.e., 3.3 mW cm^−2^ during 15 min). The irradiance was determined using an optical power meter (Newport, 1916R) coupled to an optical detector (Newport, 818‐UV).

Twenty‐4 h after irradiation, cell viability was assessed using the MTT assay by adding 50 µL of MTT solution (1 mg mL^−1^) to each well, followed by incubation for 3 h at 37 °C [36]. Formazan crystals were solubilized with 100 µL of DMSO, and absorbance was measured at 570 nm using a microplate reader (Multiskan GO, Thermo Scientific).

Cell viability was also evaluated using the NR uptake assay under identical PDT experimental conditions. After treatment, cells were incubated with 100 µL of NR solution (40 µg mL^−1^) for 2 h at 37 °C. The dye was then removed, cells were washed with PBS, and the internalized dye was solubilized using 150 µL of a decolorizing solution (50% ethanol, 49% Milli‐Q water, and 1% glacial acetic acid) under constant agitation and protected from light [37].

Cell viability, determined by either MTT or NR assays, was expressed as a percentage relative to untreated control cells according to Equation ((3))

(3) $$\%\text{Viable Cells}=\frac{{\mathrm{Abs}}_{\text{treatment}}}{\text{Average}\;{\mathrm{Abs}}_{\text{control}}}\times 100\%$$

where Abs_treatment_ is the absorbance measure at 540 nm for the neutral red (NR) assay or 570 nm for the MTT assay with cells treated with PSs and light or only with PSs in the absence of light (dark control), and AverageAbs_control_ corresponds to the absorbance at 540 nm for NR or 570 nm for MTT in untreated control cells (with or without light exposure). All experiments were performed in independent duplicate, with four technical repetitions per group (*n* = 8). Data were expressed as mean ± standard deviation (SD).

### 2.7 Detection of Reactive Oxygen Species (ROS) Using DCFH‐DA

Porphyrins were initially solubilized in DMSO and diluted in complete culture medium to a final concentration of 300 nM, ensuring a final DMSO content of less than 1% (v/v). MDA‐MB‐231 cells were seeded in black 96‐well plates at a density of 2.5 × 10^4 ^cells per well and incubated for 24 h at 37 °C in a humidified atmosphere containing 5% CO_2_ to allow cell adhesion.

After this period, the culture medium was removed and replaced with fresh complete medium containing the photosensitizer (300 nM), followed by incubation for 24 h in the dark. At the end of the PS incubation period, cells were washed with PBS (pH 7.4) to remove noninternalized photosensitizer, and 100 µL of PBS was added to each well.

Cells were then irradiated using a white LED positioned 7 cm above the plate, irradiated with 3.3 mW cm^−2^ for 15 min, resulting in a light dose of 3 J cm^−2^. Immediately after irradiation, the DCFH‐DA probe (10 µM), previously diluted in serum‐free culture medium, was added to each well [38]. Cells were incubated with the probe for 25 min at 37 °C under 5% CO_2_ and protected from light. Subsequently, cells were washed with PBS to remove excess of noninternalized probe, 100 µL of PBS was added to each well, and fluorescence was measured using a microplate reader (${\text{λ}}_{\mathrm{exc}}\text{=488 nm}$; ${\text{λ}}_{\mathrm{em}}\text{=538 nm}$) [38].

### 2.8 Protein Extraction and Quantification

Porphyrins were solubilized in DMSO and diluted in complete culture medium to a final concentration of 60 nM (<1% DMSO, v/v). MDA‐MB‐231 cells were seeded in two 6‐well plates at a density of 2.0 × 10^5 ^cells per well and incubated for 48 h at 37 °C in a 5% CO_2_ atmosphere. The culture medium was then replaced with fresh complete medium containing the photosensitizer (60 nM), followed by incubation for 24 h.

After 24 h of PS incubation, cells were washed with PBS (pH 7.4) to remove noninternalized photosensitizer, and 2 mL of PBS was added per well. Irradiation was performed using a white LED (3.3 mW cm^−2^) positioned 7 cm above the plate for 15 min, delivering a light dose of 3 J cm^−2^. Following irradiation, complete culture medium was added, and cells were incubated for 24 h post‐irradiated at 37 °C and 5% CO_2_.

Cells were then washed with PBS and lysed by adding 1 mL of ice‐cold PBS–KCl buffer (20 mM phosphate, 140 mM KCl, pH 7.4). Cells were detached using a cell scraper, collected into microcentrifuge tubes, and subjected to three freeze–thaw cycles using liquid nitrogen and a 45 °C water bath to ensure complete lysis. Total protein content in the samples was determined using the Bradford method, using an analytical curve prepared with bovine serum albumin as the standard [39].

### 2.9 Quantification of Total Thiol Content

Free thiol groups were quantified according to the method described by Ellman [40], using DTNB as the analytical reagent. Samples of lysed cells (15 µL) were diluted in 250 µL of PBS (pH 7.4), followed by the addition of 10 µL of DTNB solution (10 mM). After incubation for 30 min at room temperature, absorbance was measured at 412 nm.

Free thiol concentration was calculated using a molar extinction coefficient of 14,150 M^−1 ^cm^−1^ [41] according to Equation ((4))

(4) $$\left[\text{Free thiols}\right]=\frac{\mathrm{Abs}\;\times \;0.275\;\times \;10^{9}}{14,150\;\times \;15}$$

where Abs corresponds to the absorbances of the samples measured at 412 nm. The factor 0.275 refers to the total volume of the reaction mixture (µL), while the factor 1 × 10^8^ corresponds to the conversion of the activity to milliunits. The value 15 represents the volume of the sample added to the well, and 14,150 M^−1^·cm^−1^ corresponds to the molar extinction coefficient of DTNB [41]. The results were expressed nmol TNB equivalents/mg of protein. The experiment was performed with three technical repetitions per group (*n* = 3). The data was expressed as mean ± SD.

### 2.10 Analysis of Advanced Oxidation Protein Products (AOPP)

AOPP levels were determined by mixing 40 µL of sample with 160 µL of 0.2 M citric acid in phosphate buffer (pH 7.4) in a 96‐well UV‐transparent microplate. After 2 min of incubation under agitation, absorbance was measured at 340 nm against a blank containing citric acid and potassium iodide.

A calibration curve was generated using chloramine‐T as a standard by combining 190 µL of chloramine‐T solution with 10 µL of 1.16 M potassium iodide. AOPP concentrations were calculated by interpolating absorbance values at 340 nm onto the calibration curve and are expressed as µM chloramine‐T equivalents [42]. Experiments were performed with three technical replicates per group (*n* = 9), and data are expressed as mean ± SD.

### 2.11 Superoxide Dismutase (SOD) Activity

SOD activity was determined based on its ability to inhibit the auto‐oxidation of pyrogallol in alkaline conditions, which generates a chromogenic product detectable at 420 nm [43]. Superoxide radicals produced during pyrogallol auto‐oxidation are scavenged by SOD, resulting in reduced formation of the oxidized product.

The assay was conducted in 96‐well microplates using a reaction buffer containing 50 mM Tris‐HCl and 1 mM EDTA (pH 8.2). Pyrogallol was freshly prepared at 24 mM in 10 mM HCl. Stock solutions of catalase (2653 U mL^−1^) and SOD (5571 U mL^−1^) were prepared and working solutions were obtained immediately prior to use. A standard SOD solution was adjusted to 10 U mL^−1^.

A calibration curve was constructed using SOD standards (0, 0.25, 0.5, and 1 U mL^−1^). Reactions were initiated by the addition of pyrogallol. Each well contained 15 µL of sample, 216 µL of reaction mixture (buffer containing catalase), and 20 µL of pyrogallol, resulting in a final volume of 251 µL. Absorbance was monitored kinetically at 420 nm for 10 min with readings every 15 s. SOD activity was calculated from the calibration curve and expressed as units per milligram of protein (U mg^−1^). Experiments were performed with three technical replicates per group (*n* = 3), and data are reported as mean ± SD.

### 2.12 Catalase Activity

Catalase activity was determined by monitoring the decomposition of hydrogen peroxide (H_2_O_2_), measured as a decrease in absorbance at 240 nm [44]. The reaction buffer consisted of 10 mM potassium phosphate buffer (pH 7.0). Hydrogen peroxide was freshly prepared in the same buffer and protected from light until use. Samples (20 µL) were incubated with 290 µL of reaction mixture in UV‐transparent microplates, and absorbance at 240 nm was recorded kinetically for 10 min at 10s intervals using a microplate reader. Samples were analyzed individually to ensure accurate monitoring of absorbance decay. Catalase activity was calculated using Equation (5)

(5) $$\mathrm{CAT}=\frac{\left[({\mathrm{Abs}}_{\text{initial}}-{\mathrm{Abs}}_{\mathrm{end}})\times 310\;\times \;1000\right]}{\text{total protein concentration}\;10\;\times \;43.6}$$

where Abs_initial_ and Abs_end_ correspond to the absorbances measured at the beginning and end of the reaction, respectively. The factor 310 refers to the total volume of the reaction mixture (µL), while the factor 1000 corresponds to the conversion of activity to milliunits. The total protein concentration (mg/mL) was used to normalize the enzymatic activity. The value 10 represents the reaction time (s) and 43.6 M^−1^·cm^−1^ corresponds to the molar extinction coefficient of H_2_O_2_ at 240 nm, considering an optical path of 1 cm. The results were expressed in units of catalase activity per milligram of protein. The experiment was performed with three technical repetitions per group (*n* = 3). The data were expressed as mean ± SD.

### 2.13 Total Antioxidant Capacity via FRAP (Ferric Reducing Antioxidant Power) Assay

Total antioxidant capacity was evaluated using the ferric reducing antioxidant power (FRAP) assay, adapted from Benzie and Strain [45]. This method is based on the reduction of ferric ions (Fe^3+^) to ferrous ions (Fe^2+^) by antioxidants present in the sample, resulting in the formation of an intense blue Fe^2+^–TPTZ complex, which is quantified spectrophotometrically.

The FRAP reagent was freshly prepared on the day of analysis by mixing 0.3 M sodium acetate buffer (pH 3.6), 10 mM TPTZ solution in 40 mM HCl, and 20 mM ferric chloride solution in a volumetric ratio of 10:1:1 (v/v/v). The reagent was protected from light until use. A Trolox calibration curve was constructed using a 10 mM Trolox stock solution, from which serial dilutions were prepared to obtain concentrations in the micromolar range. A blank containing only buffer and FRAP reagent was included for absorbance correction.

Samples were analyzed in triplicate. For each well, 10 µL of sample, 25 µL of assay buffer, and 250 µL of freshly prepared FRAP reagent were added. After homogenization, plates were incubated at 37 °C for 6 min, and absorbance was measured at 593 nm using a microplate reader. Sample absorbance values were corrected using the blank and interpolated from the Trolox standard curve [46]. The experiment was performed with three technical repetitions per group (*n* = 3). The data was expressed as mean ± SD.

### 2.14 Mitochondrial Depolarization (Δ*Ψ*_m_) via Flow Cytometry

Porphyrins were solubilized in DMSO and diluted in complete culture medium to a final concentration of 60 nM, ensuring a final DMSO content of less than 1% (v/v). MDA‐MB‐231 cells were seeded in 12‐well plates at a density of 2.0 × 10^5^ cells per well and incubated for 24 h at 37 °C in a humidified atmosphere 5% CO_2_. After cell adhesion, the culture medium was replaced with fresh medium containing the photosensitizer (60 nM), followed by incubation for 3 h in the dark.

After incubation, cells were washed with PBS (pH 7.4) to remove noninternalized photosensitizer. PBS (1 mL) was added, and irradiation was performed using a white LED positioned 10 cm above the plate, delivering an irradiance of 9.8 mW cm^−2^ for 7 min, resulting in a light dose of 4 J cm^−2^. The PBS was collected, and complete culture medium was added to the wells. Cells were allowed to recover for 30 min at 37 °C and 5% CO_2_ to enhance detection of mitochondrial depolarization [47].

Cells were then harvested by trypsinization (0.4 mL of trypsin for 5 min), neutralized with complete medium, and combined with cells collected from the supernatant. Samples were centrifuged at 1500 rpm for 5 min at room temperature, washed with PBS, and centrifuged again at 700 rpm for 5 min. Cell pellets were resuspended and incubated with Rhodamine 123 (500 nM) for 10 min at room temperature, except for the unstained control.

Following incubation, cells were washed twice with PBS to remove excess dye and resuspended in 300 µL of PBS/EDTA. Fluorescence was analyzed using a BD FACSCanto II flow cytometer (excitation at 488 nm; emission collected using a 530/30 nm filter). Data was analyzed using the FlowJo software.

For each sample, at least 15,000 events were acquired. The same gating strategy was applied to all samples using control samples marked with Rhodamine 123. Representative gating plots and fluorescence histograms are provided in Figure S1. Individual median fluorescence intensity values used are provided in Table S1.

### 2.15 Activation of Caspase‐3 After Photosensitization

MDA‐MB‐231 cells were seeded in 6‐well plates at a density of 2.0 × 10^5^ cells per well and incubated for 24 h at 37 °C and 5% CO_2_. After cell adhesion, the culture medium was replaced with fresh complete medium containing the photosensitizer (60 nM), followed by incubation for 24 h. Cells were then washed with PBS to remove noninternalized photosensitizer, and 2 mL of PBS was added per well. Irradiation was performed using a white LED (3.3 mW cm^−2^) positioned 7 cm above the plate for 15 min, corresponding to a light dose of 3 J cm^−2^. Immediately after irradiation, complete culture medium was added, and cells were incubated for an additional 24 h post‐irradiation at 37 °C and 5% CO_2_.

Following incubation, cells were washed with PBS and lysed by adding 50 µL of lysis buffer (50 mM Tris‐HCl, 150 mM NaCl, 0.5% Triton X‐100) supplemented with protease inhibitors (1 mM phenylmethanesulfonyl fluoride and 1 mM sodium orthovanadate). Plates were stored around −18 °C for 24 h. Cell lysates were collected by scraping, centrifuged at 14,000 rpm for 15 min at 4 °C, and the supernatants were transferred to fresh tubes.

Cleaved caspase‐3 activity was quantified using the EnzChek Caspase‐3 Assay Kit #1 (Z‐DEVD‐AMC substrate, E‐13,183), following the manufacturer's instructions. Aliquots (50 µL) of each sample (*n* = 3) were mixed with reaction buffer (50 mM 1,4‐piperazinodiethanesulfonic acid (PIPES), pH 7.4, 10 mM ethylenediaminetetraacetic acid (EDTA), 0.5% 3‐[(3‐cholamidopropyl)dimethylammonio]‐1‐propanesulfonate (CHAPS), (±)‐ *threo* −1,4‐dimercapto‐2,3‐butanediol (DTT), and tetrapeptide sequence Asp‐Glu‐Val‐Asp (DEVD) coupled to an AMC group and protected by the Z group (Cbz, benzyloxycarbonyl) (Z‐DEVD‐AMC) in a 1:1 ratio and incubated in the dark for 30 min at room temperature. Fluorescence was measured using a microplate fluorimeter (*λ*_exc_ ≈ 342 nm; *λ*_em_ ≈ 441 nm), and active caspase‐3 levels were normalized to total protein concentration [48].

### 2.16 Glutathione (GSH) and Oxidized Glutathione (GSSG)

The levels of reduced glutathione (GSH) and oxidized glutathione (GSSG) were evaluated. GSH and GSSG contents were determined using a kinetic assay [49]. Briefly, cells were solubilized in extraction buffer (0.1% Triton X‐100 and 0.6% sulfosalicylic acid in 0.1 M potassium phosphate buffer (KPE) containing 5 mM disodium EDTA). After freeze/thaw cycles, cells were centrifuged at 3000 × g for 4 min at 4 °C, and the supernatant was used in the assay. Samples were added to a 96‐well microplate, followed by a working mixture (KPE, DTNB, and glutathione reductase) and NADPH, and absorbance was read at 405 nm every 30 s for 2 min. For GSSG determination, 2 μL of 2‐vinylpyridine (1:10) was added to 100 μL of the cell homogenate, followed by the addition of 4 μL of triethanolamine (1:6) after 1 h of incubation; subsequently, the derivatized samples were assayed as described above. Reduced and oxidized glutathione salts were used to construct the standard curves. All analyses were performed in triplicate [50]. Results were normalized to protein concentration, determined by the Bradford method [39], and expressed as units per mg of protein (U/mg protein).

### 2.17 Statistical Analysis

Statistical analyses and graphical representations were performed using the GraphPad Prism 8.0 software. All analyses were performed in triplicate (*n* = 3), and the data were presented as mean ± SD. Differences were assessed using one‐way and two‐way ANOVA, as appropriate, followed by Tukey's multiple comparisons test to compare treated groups with the control. A *p* value <0.05 was considered statistically significant.

## 3 Results and Discussion

### 3.1 PpIX Derivatives Enhance the Photosensitizer Lipophilicity

Lipophilicity is an important parameter in the molecular design process of new photodynamic compounds, since both excessively low and high Log *p* values may compromise cellular uptake, subcellular localization, and, consequently, phototherapeutic efficacy. The *n*‐octanol/water partition coefficient (Log P), widely accepted as a predictor of membrane permeability, reflects the balance between the hydrophilic and hydrophobic domains of a molecule, with values between 1 and 4 generally favoring transcellular transport and intracellular accumulation [19, 51, 52]. This parameter is also used in conjunction with other physicochemical and photophysical properties—such as the molar extinction coefficient and singlet oxygen quantum yield—to guide the rational development of PS with optimized performance.

**FIGURE 2:**
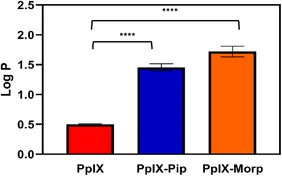
Log *p* values of PpIX, PpIX‐Pip and PpIX‐Morp. Data represent mean ± SD (*n* = 3). Statistical analysis was performed using one‐way ANOVA followed by Tukey's multiple comparisons test. Significant differences are indicated as **** *p* < 0.0001.

In this context, the Log P of each PpIX derivative was determined by using the shake‐flask method in the *n*‐octanol/water system, to better understand the lipophilicity profile of each structure and its possible relationship with cellular uptake and photodynamic performance. The values obtained are presented in Figure 2.

The incorporation of the piperazine (Pip) moiety resulted in an approximately 2.9‐fold increase in Log P compared to unmodified PpIX, whereas incorporation of the morpholine (Morp) moiety led to an increase of about 3.4‐fold (Figure 2). Furthermore, comparison between the modified derivatives showed that PpIX‐Morp exhibited a Log P approximately 1.2‐fold higher than of PpIX‐Pip (Figure 2). Therefore, the synthesized derivatives exhibit enhanced lipophilicity relative to unmodified PpIX and present Log *p* values within the range of 1–4, which may favor their ability to cross biological membranes [51].

### 3.2 PpIX‐Pip and PpIX‐Morp Improved Cellular Uptake in Breast Cancer Cells

**FIGURE 3:**
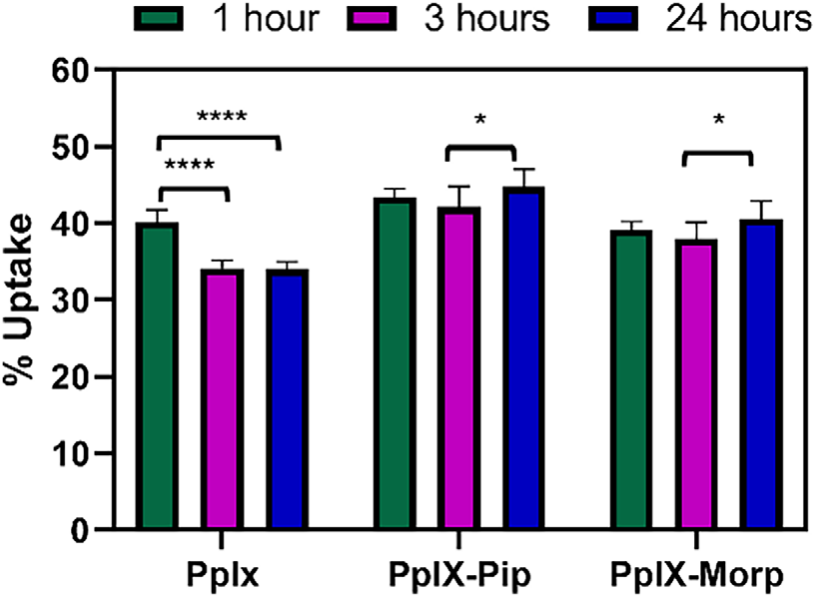
Cellular uptake percentage of PpIX, PpIX‐Pip, and PpIX‐Morp (5 µM) as a function of incubation time (1, 3, and 24 h) in MDA‐MB‐231. The bars represent the mean ± SD of two independent experiments with four replicates in each experiment (*n* = 8). Statistical analysis was performed using two‐way ANOVA followed by Tukey's multiple comparisons test. Significant differences are indicated as ****p < 0.001.

To exhibit cytotoxicity, the PS must cross the plasma membrane, which primarily contain lipids and membrane proteins that regulate the entry and exit of substances in the cell [53]. In the present study, the percentage of porphyrin incorporation in MDA‐MB‐231 cells was determined from the maximum absorption of the Soret band of the porphyrins (i.e., 401 nm) (Figure 3).

At 1 h of incubation, the % incorporation values were similar among the molecules (40.2% for PpIX, 43.4% for PpIX‐Pip, and 39.2% for PpIX‐Morp), indicating that structural differences do not yet significantly influence cellular uptake. As the incubation time increased, the profiles began to diverge. A slight reduction in % incorporation was observed for PpIX, reaching 34.1% after 24 h, while the derivatives PpIX‐Pip and PpIX‐Morp maintained high and nearly constant values throughout the period (≈42%–45% for PpIX‐Pip and 38%–40% for PpIX‐Morp). This specific decrease in PpIX suggests the occurrence of cellular efflux, a phenomenon previously described for PpIX [54, 55]. On the other hand, the sustained % incorporation over time observed in the PpIX derivatives suggests that structural modifications with Pip and Morp fragments may minimize efflux processes, promoting greater intracellular retention. Although PpIX‐Morp exhibits a higher logP, PpIX‐Pip showed slightly higher uptake, suggesting that lipophilicity alone is insufficient to explain cellular behavior and that cellular uptake is determined by multiple physicochemical and biological factors including molecular size, conformational flexibility, aggregation behavior in biological media, membrane affinity, and interactions with membrane transport processes. Because these parameters were not directly evaluated, the present results support an association between structural modification and cellular retention but do not establish lipophilicity as the sole determinant of uptake.

### 3.3 PpIX Derivatives Exhibited Enhanced Phototoxic Effects in Breast Cancer Cells

The cytotoxicity and phototoxicity of the porphyrins were evaluated in human breast cell lines MDA‐MB‐231 (tumor) and MCF‐10A (nontumor) using MTT and neutral red (NR) assays. The cell studies were carried out with 24 h of PS incubation and white LED irradiation, providing a light dose of 3 J cm^−2^. The structured analysis of the results is presented in the following.

**FIGURE 4:**
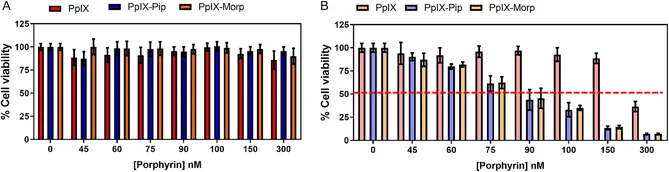
Cell viability (%) of the MDA‐MB‐231 cell line as a function of the concentration (nM) of PpIX, PpIX‐Pip and PpIX‐Morp (A) in the dark and (B) irradiated using white LED (light dose of 3 J cm^−2^). Cell viability was determined by the MTT method at 24 h post‐irradiation. Data are presented as mean ± SEM from two independent biological experiments, each performed with four technical replicates wells per condition, resulting in *n* = 8.

Figure 4 shows cell viability as a function of concentration (45–300 nM) for PpIX, PpIX Pip, and PpIX‐Morp after 24 h of incubation under dark conditions and upon white LED irradiation. In the dark, none of the compounds showed significant cytotoxicity, maintaining cell viability greater than 85% even at the highest concentrations evaluated (Figure 4A), indicating low intrinsic cytotoxicity and adequate photodynamic selectivity [56]. This behavior indicates that the insertion of Pip and Morp fragments in PpIX does not increase basal toxicity, which is an essential characteristic for photosensitizers, whose therapeutic action should strictly depend on light activation.

As observed in Figure 4B, cell viability decreased significantly after light activation, showing a concentration‐dependent effect, which confirms the photodynamic behavior of the analyzed compounds. The derivatives PpIX‐Pip and PpIX‐Morp exhibited more pronounced phototoxicity than PpIX, promoting a significant reduction in cell viability at concentrations around 75 nM, while PpIX demonstrated a photodynamic effect only at concentrations above 150 nM. After irradiation, PpIX presented an IC_50_ value of approximately 268 nM (Table 1). These values are consistent with previously reported data for breast cancer cells, in which PpIX—usually accumulated from the precursor 5‐aminolevulinate (5‐ALA)—induces significant cytotoxicity after photoactivation with a dose of 1.5, 3, 6, 9 and 12 J cm^−2^, using concentration 1 mM, primarily through the generation of reactive oxygen species and oxidative damage to essential cellular components [57, 58, 59]. The nanomolar concentrations observed in this study are consistent with the photodynamic efficacy described for PpIX [33], considering differences in experimental conditions between studies.

**TABLE 1:** IC_50_ (nM) values for PpIX, PpIX‐Pip, and PpIX‐Morp in the absence or presence of light irradiation at 3 J cm^−2^ in the MDA‐MB‐231 cell line, determined using the MTT and NR methods at 24 h post‐treatment.

**IC**_**50,**_ **nM in MDA‐MB‐231 cells**				
Method	MTT		NR	
Condition	Dark	Irradiated	Dark	Irradiated
**PpIX**	>300	267.93 ± 4.36	>300	215.02 ± 26.74
**PpIX‐Pip**	>300	86.62 ± 1.92	>300	54.70 ± 10.50
**PpIX‐Morp**	>300	85.56 ± 1.92	>300	73.31 ± 0.94

The PpIX‐Pip and PpIX‐Morp derivatives showed IC_50_ values approximately 3.1‐fold lower than unmodified PpIX (Table 1). This improved photoactivity may be related to their higher lipophilicity (Figure 2) and enhanced cellular retention after 24 h of incubation, as previously demonstrated (Figure 3). Thus, structural modifications with piperazine and morpholine conferred significant gains in photodynamic activity, making the derivatives substantially more effective than the original PpIX.

In order to avoid possible biases associated with the MTT assay and increase the robustness and reliability of experimental data, the cytotoxicity and photodynamic effect of PSs were also evaluated using the neutral red (NR) uptake assay (Figure S2). This method estimates cell viability based on the functional integrity of lysosomes [60, 61].

Thus, the results obtained in the NR assay were consistent with those observed in the MTT assay, showing the same phototoxicity trend between the compounds. In both assays, PpIX‐Pip and PpIX‐Morp exhibited significantly lower IC_50_ values compared to PpIX under irradiation conditions (Table 1). Specifically, in the MTT assay, the IC_50_ values were approximately 3.1‐fold lower for PpIX‐Pip and PpIX‐Morp compared to PpIX. Similarly, in the NR assay, the IC_50_ values were reduced by approximately 3.9‐fold for PpIX‐Pip and 2.9‐fold for PpIX‐Morp. These findings indicate that both methodologies provide concordant and complementary results, reinforcing that the structural modification of PpIX with piperazine and morpholine groups increases its photodynamic efficiency compared to the original compound.

It is important to note that the lower cellular IC_50_ values of PpIX‐Pip and PpIX‐Morp cannot presently be assigned to should not be attributed to a single parameter, such as enhanced cellular uptake or singlet oxygen quantum yields (Φ_Δ_). Photodynamic performance can also depend on aggregation [33], excited‐state behavior, ROS‐generation pathways, intracellular distribution, and susceptibility of the affected cellular targets.

In order to evaluate the selectivity of the photosensitizers, cytotoxicity and phototoxicity assays were performed in normal breast cells (MCF‐10A). Similar to what was observed for MDA‐MB‐231, no toxicity in the dark was verified for any of the compounds (Figure S3). After 24 h of incubation of the PS and irradiation using white LED at 3 J cm^−2^, a reduction in cell viability was observed for all PS (Figure S3), indicating an absence of tumor selectivity under the evaluated conditions. The IC_50_ values were comparable to those obtained for MDA‐MB‐231 cells, with only slight variation (Table 2).

**TABLE 2:** IC_50_ (nM) values for PpIX, PpIX‐Pip, and PpIX‐Morp the absence or presence of light irradiation at 3 J cm^−2^ in the MCF‐10A cell line, determined by the MTT method at 24 h post‐treatment. The selectivity index (SI) was calculated as IC_50_ (irradiated nontumor cells)/IC_50_ (irradiated MDA‐MB‐231 cells).

**IC**_**50**_ **(nM) in MCF‐10A cells**					
Method		MTT			
Condition		Dark	Irradiated	Selectivity index (SI)	
**PpIX**		>300	382.0 ± 131.0	1.42	
**PpIX‐Pip**		>300	91.01 ± 16.05	1.05	
**PpIX‐Morp**		>300	77.42 ± 26.58	0.90	

The IC_50_ values measured in MCF‐10A cells were close to those obtained in MDA‐MB‐231 cells, resulting in selectivity indices of 1.42 for PpIX, 1.05 for PpIX‐Pip, and 0.90 for PpIX‐Morp (Table 2). Thus, under the present *in vitro* conditions, the derivatives did not exhibit preferential phototoxicity toward MDA‐MB‐231 tumor cells. In particular, an SI close to 1 indicates comparable sensitivity of the two cell lines, whereas the value below 1 obtained for PpIX‐Morp indicates that no tumor‐selective advantage was detected.

While evaluating both tumoral and nontumoral cell lines is standard practice to demonstrate the specificity of conventional anticancer agents, the paradigm differs significantly for photodynamic therapy (PDT). True PDT selectivity relies on two main pillars: negligible toxicity in the absence of light (as demonstrated in Figures 4, S2, and S2) and the spatial confinement of light irradiation. Still, the mechanism of PDT relies on the generation of ROS by PS only upon light exposure. The relatively small difference between the IC_50_ values obtained for MDA‐MB‐231 and MCF‐10A cells likely reflects the fact that PDT activity depends primarily on ROS generation during light activation rather than on tumor‐specific molecular targets.

Unlike conventional cytotoxic agents, whose selectivity intrinsically depends on specific biochemical mechanisms, the therapeutic efficacy of PDT is ultimately achieved through the combination of absence of toxicity in dark, spatial application of light irradiation and preferential PS accumulation in tumor. Since PS remains nontoxic in the absence of light, selectivity can be achieved by controlling the tissue´s irradiated area in PDT context [7]. Thus, the lack of cellular selectivity observed in this study does not preclude the phototherapeutic potential of these compounds. The PpIX derivatives investigated here remain promising candidates for photodynamic applications.

### 3.4 Detection of Reactive Oxygen Species (ROS) Using the DCFH‐DA Probe

**FIGURE 5:**
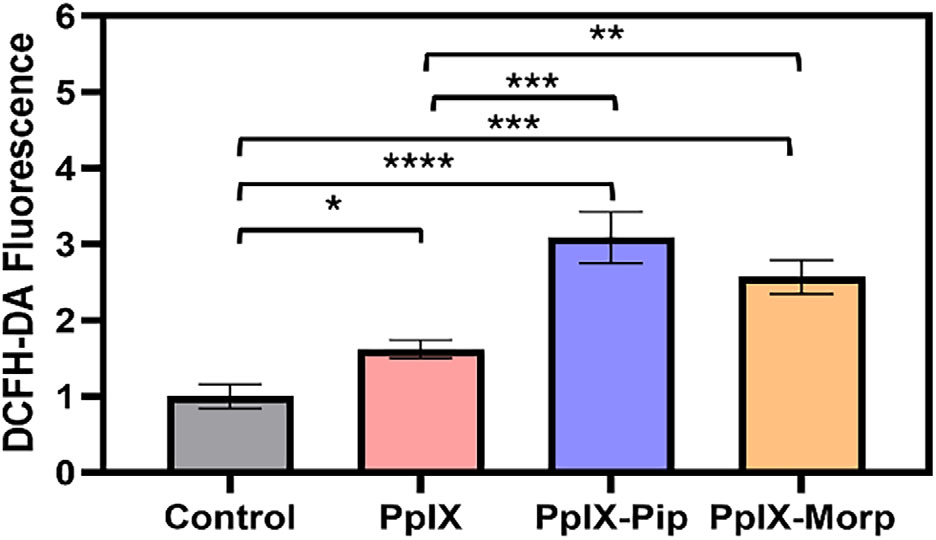
Relative DCFH‐DA fluorescence to measure general ROS formation immediately after irradiation (white LED at 3 J cm^−2^) using PpIX, PpIX‐Pip, and PpIX‐Morp at 300 nM in the MDA‐MB‐231 cell line. The bars represent the mean ± SD of an experiment, each with three replicates (*n* = 3). Statistical analysis was carried out using one‐way ANOVA followed by Tukey's multiple comparisons test, comparing treated groups to control. Significant differences are indicated as * *p* < 0.05 and **** *p* < 0.0001.

The 2′,7′‐dichlorodihydrofluorescein diacetate (DCFH‐DA) assay was used to evaluate ROS production after photoactivation of PS in cells, with ROS levels estimated by fluorescence intensity. The results are presented in Figure 5 for incubation with PS for 24 h followed by irradiation with a dose of 3 J cm^−2^.

All photosensitizers induced a significant increase in ROS generation compared to the control (Figure 5). It is noted that, again, the PpIX derivatives generated substantially higher levels of ROS compared to the PpIX precursor (Figure 5). These results are consistent with the higher phototoxicity observed for PpIX‐Pip and PpIX‐Morp relative to PpIX (Figures 4 and S2).

### 3.5 Oxidative Stress Is Increased After Photosensitization of MDA‐MB‐231 Cells

The combined analysis of biomolecule (lipids and proteins) oxidation and cellular antioxidant response after photodynamic therapy revealed consistent differences between PpIX and its derivatives.

**FIGURE 6:**
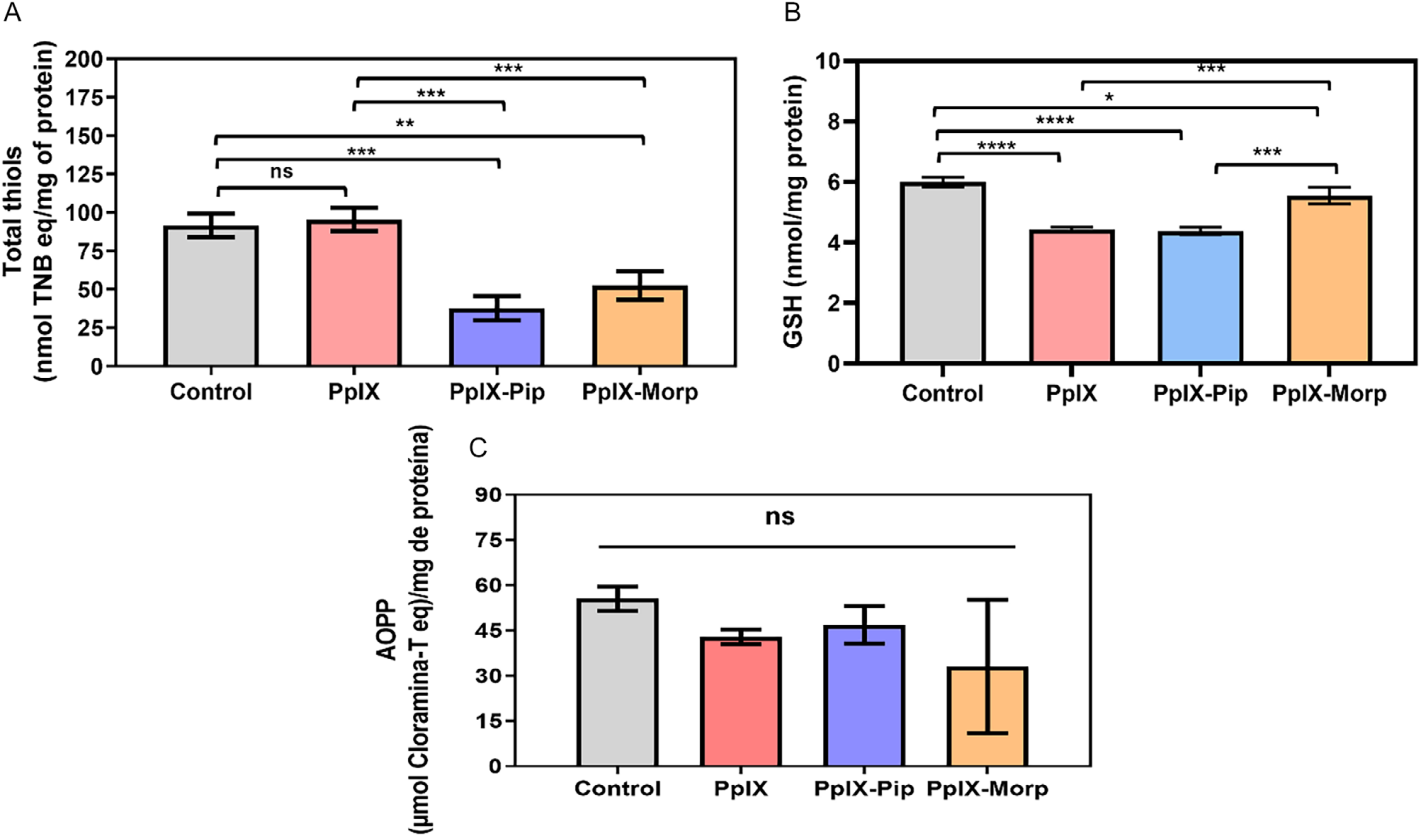
Oxidative stress‐related parameters evaluated 24 h after photosensitization of MDA‐MB‐231 cells treated with 60 nM of PpIX, PpIX‐Pip, and PpIX‐Morp under white LED irradiation (3 J cm^−2^). Control refers to cells in the absence of photosensitizer (PS). (A) Total thiol content, (B) total glutathione (GSH) levels, and (C) levels of advanced oxidative protein products (AOPPs). Data are presented as mean ± SD (*n* = 3). Statistical analysis was performed using one‐way ANOVA followed by Tukey's multiple comparisons test, comparing treated groups with the control. Statistical significance is indicated as ns, not significant and ***p < 0.0001.

Quantification of sulfhydryl groups by the DTNB assay indicated a reduction in intracellular thiol levels in cells treated with PpIX‐Pip and PpIX‐Morp under irradiation, suggesting the occurrence of initial protein oxidation, while PpIX did not show statistically relevant changes compared to the control (Figure 6A).

In agreement with these findings, intracellular antioxidant status was further compromised, as evidenced by a reduction in glutathione (GSH) levels (Figure 6B). GSH depletion suggests its consumption during ROS detoxification, reinforcing the presence of oxidative stress. Together with the observed decrease in total thiols and FRAP values (Figure S4), these results indicate that nonenzymatic antioxidant defenses are actively engaged but become progressively depleted following treatment with PpIX derivatives.

Results from the advanced protein oxidation products (AOPP) analysis showed no significant increase, indicating that, under the experimental conditions employed, the oxidative damage remained restricted to the initial stages and did not evolve into irreversible modifications detectable by this method (Figure 6C).

**FIGURE 7:**
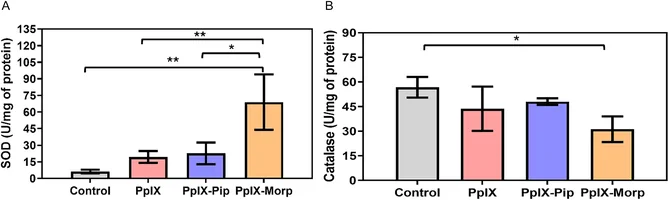
Enzymatic antioxidant response, 24 h after photosensitization of MDA‐MB‐231 cells treated with 60 nM of PpIX, PpIX‐Pip, and PpIX‐Morp under white LED irradiation (3 J cm^−2^). Control refers to cells in absence of PS treatment. (A) Superoxide dismutase (SOD) activity and (B) Catalase (CAT) activity. Statistical analysis was carried out using one‐way ANOVA followed by Tukey's multiple comparisons test, comparing treated groups to control. Statistical significance is indicated as * *p* < 0.05 and ** *p* < 0.005.

Regarding the enzymatic antioxidant response, only cells photosensitized with PpIX‐Morp exhibited statistically significant changes in SOD and catalase activities, relative to the control (Figure 7). PpIX‐Morp induced a significant increase in SOD activity (Figure 7A) and a significant decrease in catalase activity (Figure 7B). These results indicate that PpIX‐Morp produced a measurable alteration in the balance between two major antioxidant enzymes under the conditions evaluated. Nevertheless, enzyme activity was measured at a single post‐irradiation time point (24 h), and the present data does not establish whether the observed changes resulted from adaptive regulation, altered enzyme abundance, or enzyme activation/inactivation. The interpretation has therefore been restricted to the statistically supported treatment effect.

Note that SOD converts superoxide into H_2_O_2_, whereas catalase contributes to H_2_O_2_ decompositionl; this enzymatic profile could favor altered peroxide homeostasis. Nevertheless, intracellular H_2_O_2_ was not directly quantified, and the present results do not demonstrate its accumulation. H_2_O_2_ may have been transiently formed and subsequently consumed, or it may have been partially removed by other antioxidant systems, such as glutathione peroxidases and peroxiredoxins [62]. The depletion of nonenzymatic defenses, specifically total thiols and glutathione (GSH), suggests that these systems rapidly intercept the accumulating H_2_O_2_ before it can cause advanced, irreversible protein oxidation (AOPP). Therefore, transient accumulation of H_2_O_2_ may not necessarily result in measurable increases in AOPP after 24 h.

Taken together, these results support the idea that the structural modifications of PpIX promote a more pronounced redox imbalance at the cellular level, characterized predominantly by initial protein oxidation and differential activation of antioxidant defenses, without evidence of accumulation of advanced oxidation products. These findings are consistent with the higher production of ROS and phototoxicity observed for the derivatives, especially for PpIX‐Morp, although extrapolation of these effects to in vivo scenarios should be done with caution.

### 3.6 Phototoxicity of PpIX Derivatives Is Related to Mitochondrial Damage

Mitochondria play a central role in maintaining bioenergetic homeostasis and regulating programmed cell death and are particularly sensitive to oxidative stress induced during photodynamic therapy. Alterations in mitochondrial membrane potential (ΔΨ_m_) are recognized as early events indicative of mitochondrial dysfunction and activation of apoptotic pathways. Previous evidence indicates that increased lipophilicity of photosensitizers may favor their interaction with mitochondrial membranes, thereby amplifying effects on ΔΨm and, consequently, the phototoxic response [63].

Based on this rationale, ΔΨ_m_ was assessed by flow cytometry using the fluorescent probe rhodamine 123 (Rh123). In viable cells, the mitochondrial membrane potential is typically between −150 and −180 mV, which promotes the selective accumulation of lipophilic cationic dyes inside the mitochondria, resulting in high fluorescence intensity [64, 65]. In contrast, mitochondrial depolarization compromises Rh123 sequestration, leading to a reduction in the fluorescent signal, an event widely associated with bioenergetic dysfunction and the initial activation of the apoptotic cascade [66, 67, 68].

**FIGURE 8:**
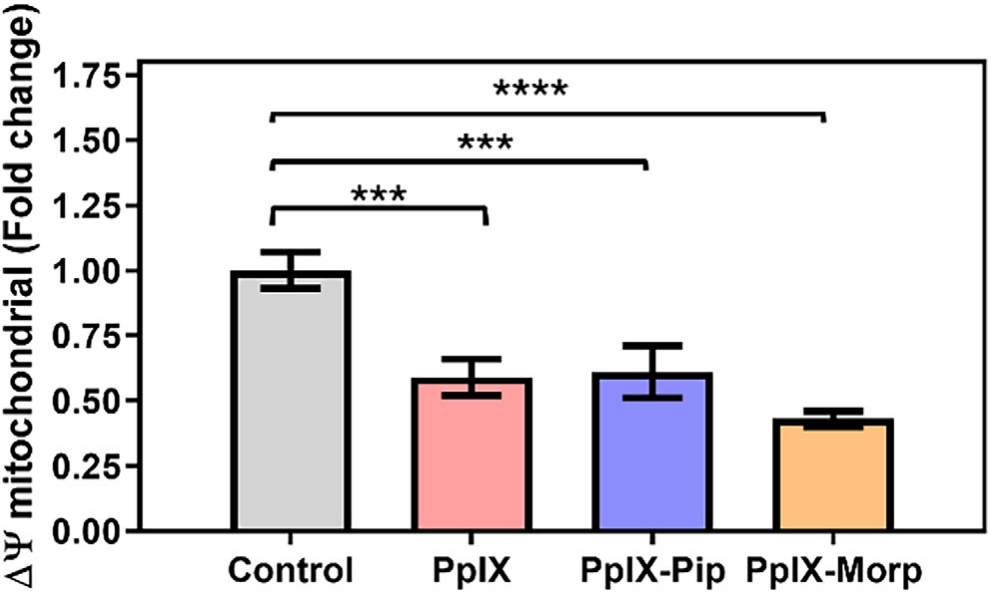
Fold change of mitochondrial membrane potential (ΔΨ_m_) depolarization in comparison to control cells. MDA‐MB‐231 cells 24 h after photosensitization with 60 nM of PpIX, PpIX‐Pip, and PpIX‐Morp under white LED irradiation (3 J cm^−2^). Control refers to cells in absence of PS treatment. Statistical analysis was carried out using one‐way ANOVA followed by Tukey's multiple comparisons test, comparing treated groups to control. Significant differences are indicated as ***p < 0.0005 and ****p < 0.0001.

Thus, the decrease in Rh123 fluorescence observed after photosensitization indicated loss of ΔΨ_m_ (Figure 8), providing further evidence that the evaluated photosensitizers affect mitochondrial integrity. These data complement the results obtained for ROS generation (Figure 5) and cellular redox imbalance (Figure 6A–C), reinforcing the involvement of mitochondrial mechanisms in induced phototoxicity, particularly for structurally modified derivatives of PpIX.

Considering the control as a reference (1.0‐fold), the relative fluorescence was reduced to approximately 0.59‐fold in the PpIX group, 0.61‐fold in the PpIX‐Pip group, and 0.43‐fold in the PpIX‐Morp group (Figure 8). Although all PpIX derivatives promote mitochondrial depolarization after photosensitization compared to control cells, no statistically significant difference was observed between PpIX, PpIX‐Pip, and PpIX‐Morp. These data show that mitochondrial dysfunction forms part of the cellular response to photosensitization but do not establish that mitochondrial depolarization is directly proportional to the total ROS signal or that the compounds preferentially localize in mitochondria.

### 3.7 PpIX‐Pip and PpIX‐Morp Trigger Apoptosis in Breast Cancer Cells After PDT

The activation of caspases‐3 constitutes a terminal event in the apoptotic pathway and is widely recognized as a marker of programmed cell death, especially in responses associated with oxidative stress and mitochondrial dysfunction. In the context of PDT, the increased levels of active caspase‐3 reflect the induction of apoptotic pathways dependent on ROS generated after photoactivation by the photosensitizer, frequently associated with the loss of mitochondrial membrane potential (ΔΨ_m_) [68, 69, 70].

**FIGURE 9:**
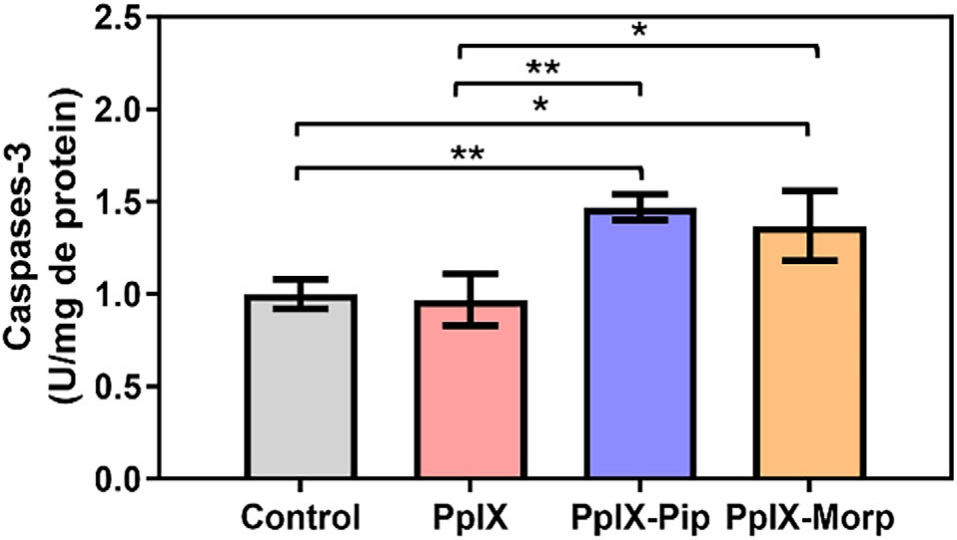
Relative caspase‐3 activity in MDA‐MB‐231 cells 24 h after photosensitization with 60 nM of PpIX, PpIX‐Pip, and PpIX‐Morp under white LED irradiation (3 J cm^−2^). Values represent the relative variation (fold change) of enzymatic activity normalized to the control group (control = 1). Statistical analysis was carried out using one‐way ANOVA followed by Tukey's multiple comparisons test, comparing treated groups to control. Significant differences are indicated as **p < 0.010 and *p < 0.05.

Cells photosensitized with PpIX showed values close to the control (Figure 9), indicating no significant caspase activation. In contrast, the PpIX‐Pip and PpIX‐Morp derivatives promoted a significant increase in active caspase‐3 content, with values of approximately 1.5‐fold, corresponding to an increase of about ≈50% compared to the control (Figure 9).

This greater caspase‐3 activation is consistent with the mitochondrial damage profile observed for these derivatives, since PpIX‐Pip and PpIX‐Morp induced marked reductions in ΔΨ_m_, with values of approximately 0.61‐fold and 0.43‐fold relative to the control, respectively (Figure 9). Furthermore, the increase in caspase‐3 activation is consistent with the other outcomes evaluated throughout the study, including increased phototoxicity (Figure 4B), increased ROS generation (Figure 5), intensified protein oxidation after photosensitization (Figure 6A), alterations in the activity of the antioxidant enzymes superoxide dismutase (Figure 7A) and catalase (Figure 7B), and a reduction in cellular antioxidant capacity (Figure S4). Taken together, these results reinforce that PpIX derivatives also promote cell death through apoptotic mechanisms mediated by mitochondrial dysfunction after PDT.

## 4 Conclusion

This study demonstrates that structural modification of protoporphyrin IX significantly influences its photodynamic performance in breast cancer cells (MDA‐MB‐321). Peripheral modification of PpIX with methylpiperazine or morpholine groups altered its lipophilicity, time‐dependent cellular retention, and photodynamic response in MDA‐MB‐231 cells. Both derivatives displayed lower light‐dependent IC_50_ values than PpIX while retaining low toxicity in the dark, indicating a clear gain in photodynamic potency without increased basal cytotoxicity. However, the relationship between molecular lipophilicity and cellular uptake was not linear, indicating that Log P alone does not explain the cellular behavior of the compounds.

Mechanistic investigations further indicated that photosensitization with the modified derivatives increased intracellular DCFH‐derived fluorescence and altered selected cellular redox parameters. The reduction of intracellular thiol levels, alterations in antioxidant enzyme activity, and decreased total antioxidant capacity collectively support the occurrence of oxidative imbalance after irradiation. In particular, PpIX‐Morp significantly increased SOD activity and decreased catalase activity, whereas no significant increase in AOPP was detected at the evaluated 24 h endpoint.

All photosensitizers altered Rh123 fluorescence, indicating disruption of mitochondrial membrane potential. The magnitude of this effect did not parallel the general intracellular oxidation signal, and the present data does not demonstrate mitochondrial localization of the compounds. Moreover, the activation of caspase‐3 suggests the involvement of mitochondria‐mediated apoptotic pathways in the phototoxic response.

Taken together, these findings highlight the importance of rational structural modification of porphyrins to modulate their biological interactions and photodynamic efficiency. The PpIX derivatives investigated here represent promising scaffolds for the development of improved porphyrin‐based photosensitizers, and the structure–activity relationships identified in this study may contribute to the design of new PDT agents with enhanced therapeutic performance.

## Funding

This study was supported by the Fundação de Amparo à Pesquisa do Estado de Minas Gerais (APQ‐02393‐24, APQ‐00864‐25, APQ‐02185‐24, RED‐00110‐23 and APQ‐02533‐23), the Conselho Nacional de Desenvolvimento Científico e Tecnológico (407282/2023‐8, 308094/2025‐5, 300079/2025‐7 and 303853/2025‐5), and the Coordenação de Aperfeiçoamento de Pessoal de Nível Superior (001, PROEX).

## Conflicts of Interest

The authors declare no conflicts of interest.

## Supporting information

Supplementary Material

## Data Availability

The data that support the findings of this study are available from the corresponding author upon reasonable request.
