# Supplementary material for: Piperazine and Morpholine Protoporphyrin IX Derivatives Intensify Biomolecules Oxidation and Phototoxicity in Triple‐Negative Breast Cancer
Source: ChemMedChem. 2026 Aug 28;21(17):e70471. doi: 10.1002/cmdc.70471 (PMC13524843; doi:10.1002/cmdc.70471)
Supplement: Supplementary file 1 [file CMDC-21-e70471-s001.pdf]

Supporting Information

Piperazine and morpholine protoporphyrin IX derivatives intensify biomolecules oxidation and phototoxicity in triple negative breast cancer

Maynne Duarte Suriani Franco[a], Thibault Joseph William Jacques Dit Lapierre[a], Tarcisio Paiva Mendonça[b], Luiz Felipe Fernandes Peixoto[c], Renata Roland Teixeira[b], Joyce Ferreira Da Costa Guerra[b], Kelly Aparecida Geraldo Yoneyama Tudini[b], Daniele Lisboa Ribeiro[c], Foued Salmen Espindola[b], Allisson Benetti Justino[b], Celso de Oliveira Rezende Júnior[a],[d], Tayana Mazin Tsubone*[a].

[a] Institute of Chemistry, Federal University of Uberlandia, Uberlandia, Minas Gerais, Brazil.
*E-mail: tayana.tsubone@ufu.br

[b] Institute of Biotechnology, Federal University of Uberlandia, Uberlandia, Minas Gerais, Brazil.

[c] Institute of Biomedical Sciences, Federal University of Uberlandia, Uberlandia, Minas Gerais, Brazil.

[d] Department of Chemistry, Federal University of Juiz de Fora, Juiz de Fora, Minas Gerais, Brazil.

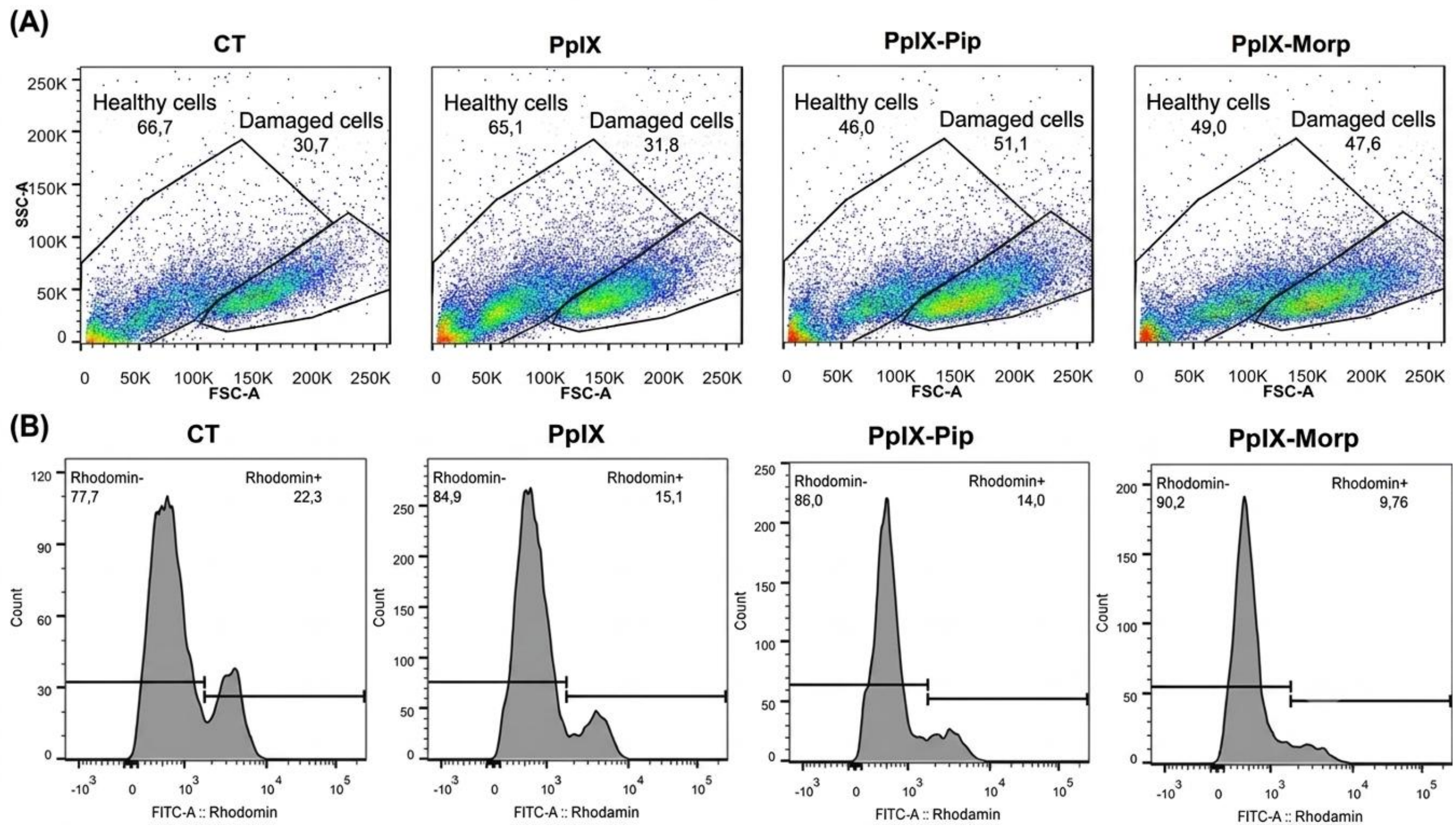

Figure S1. Flow cytometry analysis of cell integrity and mitochondrial membrane potential in MDA-MB-231 cells after photodynamic treatment with PpIX, PpIX-Pip, or PpIX-Morp. (A) Representative FSC-A versus SSC-A dot plots showing the distribution of healthy and damaged cell populations in untreated cells (CT) and treated cells. The percentages of cells within each gated population are indicated. (B) Representative histograms of Rhodamine 123 fluorescence (FITC-A) showing Rhodamine-negative and Rhodamine-positive cell populations, corresponding to cells with reduced and preserved mitochondrial membrane potential, respectively. The percentages of each population are indicated. Cells were treated with 60 nM of each photosensitizer and irradiated with white LED light (4 J cm^{-2}) before flow cytometric analysis.

Table S1. Raw median fluorescence intensity values obtained from the Rh123 mitochondrial membrane potential assay. Cells were treated with PpIX, PpIX-Pip, or PpIX-Morp under the experimental conditions described in the Materials and Methods. Median fluorescence intensity values were obtained from three independent technical replicates and were used for the calculation of the percentage of cells with polarized ($Rh123^{+}$).

Sample	Replicate 1	Replicate 2	Replicate 3	Mean ± SD
Control ($Rh123^{+}$)	1.07	0.93	-	1.00 ± 0.07
PpIX ($Rh123^{+}$)	0.63	0.49	0.64	0.59 ± 0.07
PpIX-Pip ($Rh123^{+}$)	0.58	0.48	0.77	0.61 ± 0.10
PpIX-Morp ($Rh123^{+}$)	0.41	0.48	0.41	0.43 ± 0.03

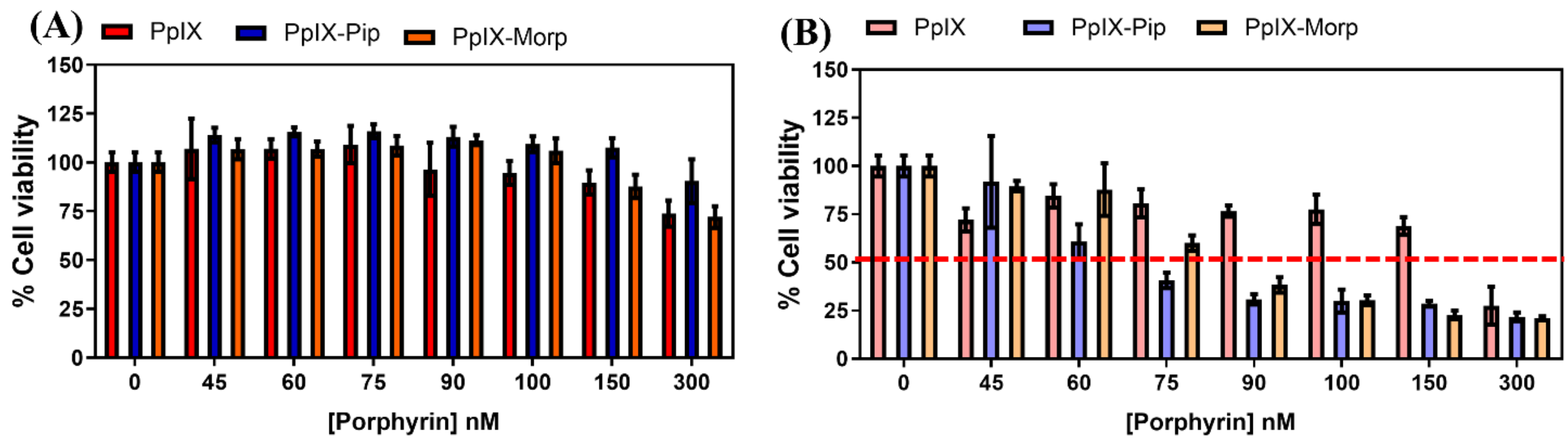

Figure S2. Cell viability (%) of the MDA-MB-231 cell line as a function of the concentration (nM) of PpIX, PpIX-Pip and PpIX-Morp (A) in the dark and (B) irradiated with white LED (light dose of 3 J cm^{-2}). Cell viability was determined by the NR method at 24 h post-irradiation. Data are presented as mean ± SEM from two independent biological experiments, each performed with four technical replicates wells per condition, resulting in n = 8.

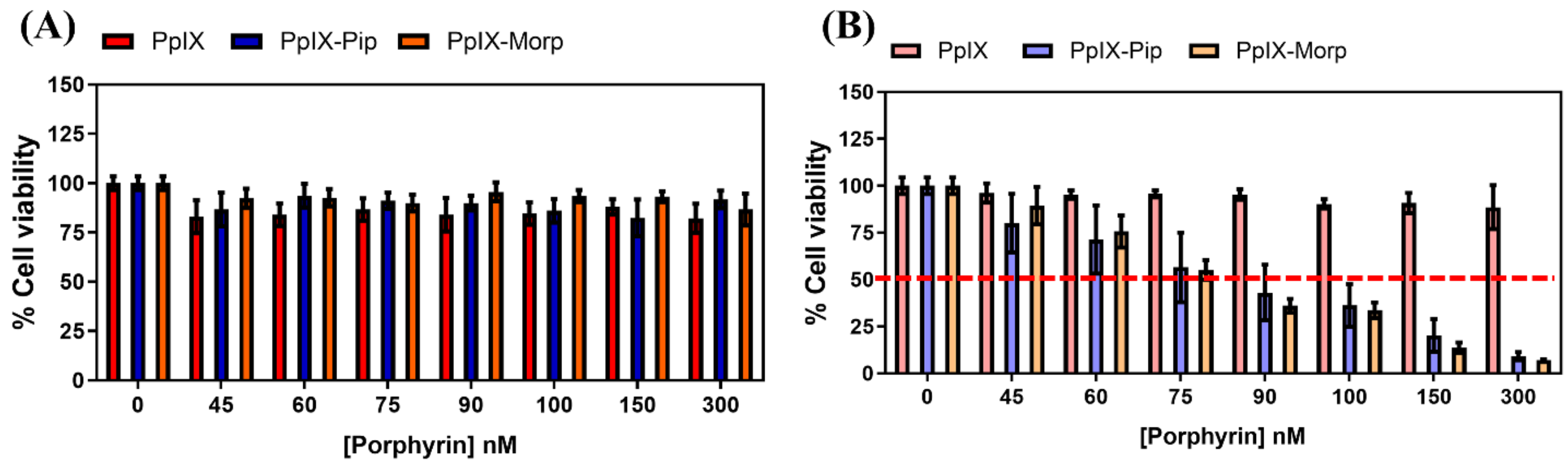

Figure S3. Cell viability (%) of the MDF-10A cell line as a function of the concentration (nM) of PpIX, PpIX-Pip and PpIX-Morp (A) in the dark and (B) irradiated with white LED (light dose of 3 J cm^{-2}). Cell viability was determined by the MTT method at 24 h post-irradiation. Data are presented as mean ± SEM from two independent biological experiments, each performed with four technical replicates wells per condition, resulting in n = 8.

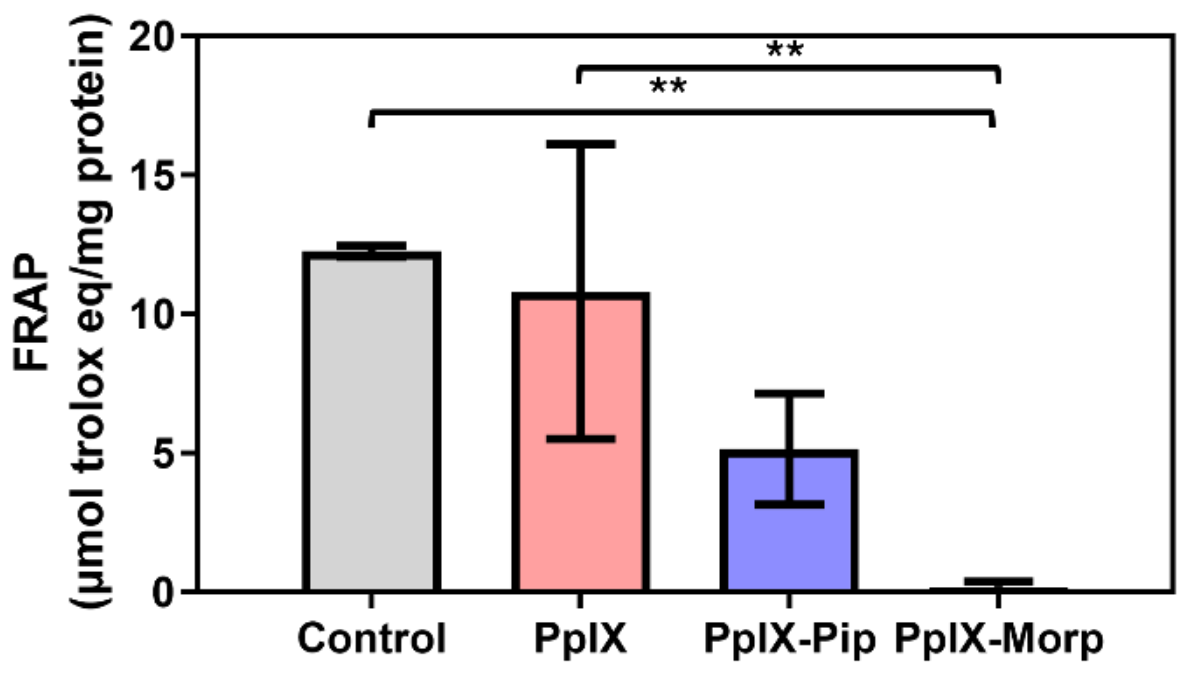

Figure S4. Cellular antioxidant response measured by FRAP assay, 24 h after photosensitization of MDA-MB-231 cells treated with 60 nM of PpIX, PpIX-Pip and PpIX-Morp under white LED irradiation (3 J cm⁻²). Control refers to cells in absence of PS treatment. Statistical analysis was carried out using one-way ANOVA followed by Tukey's multiple comparisons test, comparing treated groups to control. Significant differences are indicated as * $p < 0.05$ and ** $p < 0.005$.

Table S2. Individual normalized caspase-3 activity values in MDA-MB-231 cells treated with PpIX and its derivatives. Fluorescence intensity was normalized to the total protein content, and relative caspase-3 activity was calculated by normalizing each value to the mean of the untreated control (CT = 1.00). Individual biological replicates and the corresponding mean ± SD are shown (n = 3).

Treatment	Protein-normalized fluorescence			Relative caspase-3 activit (mean ± SD)
	Replicate 1	Replicate 2	Replicate 3	
Control	131.81	151.61	152.11	1.00 ± 0.08
PpIX	136.72	162.46	121.55	0.97 ± 0.14
PpIX-Pip	201.50	219.08	216.35	1.47 ± 0.07
PpIX-Morp	169.75	202.78	223.34	1.37 ± 0.19
